# Plant natriuretic peptides induce proteins diagnostic for an adaptive response to stress

**DOI:** 10.3389/fpls.2014.00661

**Published:** 2014-11-26

**Authors:** Ilona Turek, Claudius Marondedze, Janet I. Wheeler, Chris Gehring, Helen R. Irving

**Affiliations:** ^1^Division of Biological and Environmental Science and Engineering, King Abdullah University of Science and TechnologyThuwal, Saudi Arabia; ^2^Drug Discovery Biology, Monash Institute of Pharmaceutical Sciences, Monash UniversityMelbourne, VIC, Australia

**Keywords:** plant natriuretic peptide, peptide hormone signaling, plant homeostasis, molecular mimicry, salt stress, reactive oxygen species

## Abstract

In plants, structural and physiological evidence has suggested the presence of biologically active natriuretic peptides (PNPs). PNPs are secreted into the apoplast, are systemically mobile and elicit a range of responses signaling via cGMP. The PNP-dependent responses include tissue specific modifications of cation transport and changes in stomatal conductance and the photosynthetic rate. PNP also has a critical role in host defense responses. Surprisingly, PNP-homologs are produced by several plant pathogens during host colonization suppressing host defense responses. Here we show that a synthetic peptide representing the biologically active fragment of the *Arabidopsis thaliana* PNP (AtPNP-A) induces the production of reactive oxygen species in suspension-cultured *A. thaliana* (Col-0) cells. To identify proteins whose expression changes in an AtPNP-A dependent manner, we undertook a quantitative proteomic approach, employing tandem mass tag (TMT) labeling, to reveal temporal responses of suspension-cultured cells to 1 nM and 10 pM PNP at two different time-points post-treatment. Both concentrations yield a distinct differential proteome signature. Since only the higher (1 nM) concentration induces a ROS response, we conclude that the proteome response at the lower concentration reflects a ROS independent response. Furthermore, treatment with 1 nM PNP results in an over-representation of the gene ontology (GO) terms “oxidation-reduction process,” “translation” and “response to salt stress” and this is consistent with a role of AtPNP-A in the adaptation to environmental stress conditions.

## Introduction

Salt regulation in vertebrates depends upon the control of sodium secretion via kidney through the process of natriuresis, which is regulated by aldosterone and natriuretic peptides (NP). The first indications that plants may possess a NP signaling system came from immunoassays on tissue extracts from Florida beauty (*Dracena godseffiana*) where antibodies against the atrial natriuretic peptide (ANP) were used to detect molecules in leaves and stems (Vesely and Giordano, 1991). Exogenous application of synthetic ANP also increased the rate of transpiration, solute flow and solute uptake in carnation and chrysanthemum (Vesely et al., 1993), and subsequent studies demonstrated that synthetic rat ANP induced stomatal opening in *Tradescantia* sp. (Gehring et al., 1996; Gehring, 1999). However, the effect appeared to involve movement of ions other than Na^+^, unlike animal systems, as Na^+^ was not required in the medium. Competition *in vitro* binding assays on isolated leaf membranes using radiolabeled ANP (Gehring et al., 1996; Suwastika et al., 2000) and detection of increases in cGMP in response to application of ANP (Pharmawati et al., 1998a, 2001) indicated that a specific ligand-receptor system may be present. Immuno-affinity chromatography was used to purify biologically active PNP immuno-analogs from a number of different species, including ivy and potato (Billington et al., 1997; Maryani et al., 2001). Exogenous application of immunoreactant PNP induced stomatal opening, activation of the membrane H^+^-ATPase, transient elevation of cGMP levels, osmoticum-dependent volume changes in protoplasts, and modulated ion fluxes across plant membranes (Pharmawati et al., 1998b, 1999, 2001; Maryani et al., 2000, 2001). Collectively, these physiological effects were consistent with localization of molecules recognized by anti-ANP antibodies by *in situ* hybridization in plant conductive tissues (Maryani et al., 2003).

**Figure 1:**
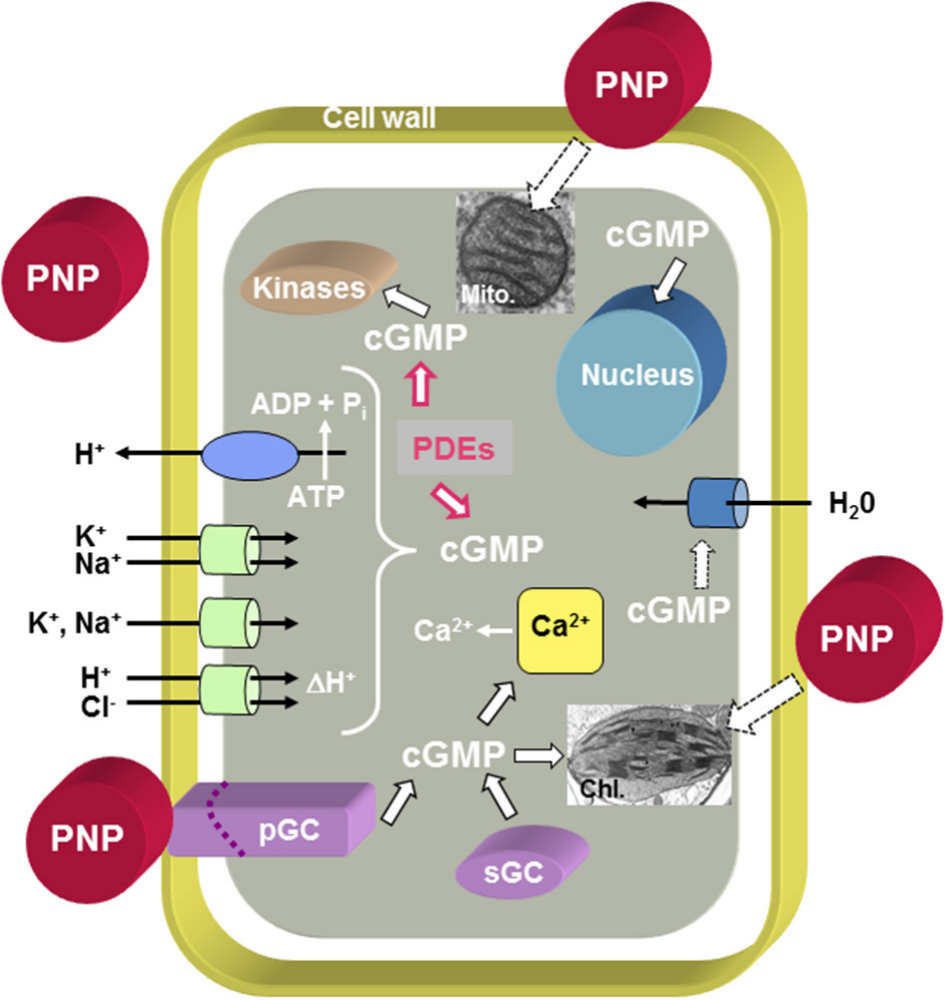
**Overview of PNP action at the cellular level**. The model proposes that PNPs can dock to receptor-like molecules with ligand-inducible guanylate cyclase activity (pGCs) and that cGMP acts as second messenger while phosphodiesterases (PDEs) metabolize cGMP to GMP. Cyclic GMP can affect cytosolic Ca^2+^ levels by modulating ion channels [e.g., Cyclic Nucleotide Gated Channels (CNGCs)], activate phosphorylation through kinases and in turn affect the transcriptome and (phospho-)proteome. It is also conceivable that PNP acts directly or indirectly via PNP-dependent phosphorylation on aquaporins. There is also evidence for PNP-dependent modulation of chloroplast and mitochondrial function. Solid black outlined arrows indicate established functions, red outlined arrows indicate inhibitory effects, and dashed outlined arrows indicate that the effects require further elucidation.

The PNP ortholog in *Arabidopsis thaliana* (AtPNP-A; GenBank Accession Number: NP_849979; TAIR: At2g18660.1) encodes a small protein of 130 amino acids (MW: 14518 kD; pI: 9.5) and orthologs of AtPNP-A and its related sequence AtPNP-B (GenBank Accession Number: NP_194767; TAIR: At4g30380.1) occur in both monocots and dicots, as well as moss (Ludidi et al., 2002; Gehring and Irving, 2003). AtPNP-A is distantly related to the N-terminal portion of the cell wall loosening expansins but it does not contain the C-terminal polysaccharide-binding domain found in expansins (Kende et al., 2004). AtPNP-A contains an N-terminal signal sequence critical for its secretion into the apoplast (Wang et al., 2011) where it has been identified in proteomic studies (Boudart et al., 2005). Purified recombinant AtPNP-A protein has been shown to stimulate guard cell opening (even modulating ABA effects on guard cells) and protoplast swelling, in addition to altering ion fluxes and generating transient increases in cGMP (Ludidi et al., 2004; Morse et al., 2004; Wang et al., 2007) (Figure 1). The active domain of AtPNP-A is the only region containing similarity to ANP (Morse et al., 2004; Wang et al., 2007) and recombinant AtPNP-A can mimic ANP in animal cells (Wang et al., 2010). The effects of PNP-A are summarized in Figure 1 and indicate that PNP-A potentially has a widespread ability to modulate intracellular responses that is as yet not properly understood at either a cellular or whole plant level.

The effects of recombinant PNP have been observed in plants where starch degradation in guard cells is rapidly stimulated along with increases in stomatal conductance and changes in photosynthetic rate, which is correlated with improved efficiency of light use during photosynthetic CO_2_ fixation (Gottig et al., 2008; Garavaglia et al., 2010b). However, recombinant AtPNP-A also induces rapid increase in dark respiration in treated leaves that is systemically spread to both upper and lower leaves by a phloem-mediated signal (Ruzvidzo et al., 2011). AtPNP-A thus appears to have roles in maintaining photosynthetic efficiency that may modify responses to stress imposed on plants. A systems level transcriptomics analysis of AtPNP-A has implicated PNP in Arabidopsis responses to both abiotic and biotic stresses (Meier et al., 2008) and transient reporter assays demonstrate that *AtPNP-A* expression is enhanced by heat, osmoticum and salt (Wang et al., 2011).

Surprisingly, various bacterial and fungal pathogens have also co-opted PNP-like molecules that they use to farm the host to their benefit. For instance, the bacterial biotrophic pathogen *Xanthomonas axonopodis* pv. *citri*, contains a gene encoding a PNP-like protein (XacPNP; GenBank Accession Number: NP_642965) (Nembaware et al., 2004) that is similar at the active region (Figure S1). Recombinant XacPNP, much like AtPNP-A, modulates photosynthesis (Gottig et al., 2008). *XacPNP* is highly induced in conditions that mimic the ionic and osmotic conditions *in planta* and reduces development of necrotic lesions in leaves while promoting bacterial cell survival much to the detriment of the host (Gottig et al., 2008). Another example involves the tomato immune receptor Ve1 that governs resistance to race 1 strains of the soil-borne vascular wilt fungi *Verticillium dahliae* and *Verticillium alboatrum*. The functional ligand Ave1 (Avirulence on Ve1) is a homolog of PNP and PNP homologs are also present in the plant pathogenic fungi *Colletotrichum higginsianum, Cercospora beticola*, and *Fusarium oxysporum* f. sp. *Lycopersici* (de Jonge et al., 2012). Since transient expression of the Ave1 homologs from *F. oxysporum* and *C. beticola* activate Ve1-mediated resistance (de Jonge et al., 2012) and Ve1 mediates resistance to *F. oxysporum* in tomato, it demonstrates that this receptor acts in resistance to several fungal pathogens.

The presence of PNP has been associated with complex and diverse functions (Figure 1) but the intracellular proteins required to mediate these processes are not known. Given that PNPs elicit a number of physiological responses and have a role in the systemic regulation of plant ion homeostasis, we have undertaken to further characterize the PNP-dependent responses at the systems level using proteomics. Since there is a complex interplay of physiological responses, we have selected a simplified cell suspension system to examine the effect of two concentrations of the active peptide fragment of PNP-A in the first instance. One of the concentrations elicits reactive oxygen species (ROS) and the second (and lower) concentration conceivably primes ROS production. The aim is to identify early changes in the proteome and then using systems tools to relate these changes to biological functions and/or pathways. Inferred PNP functions can then be used to inform detailed structural and functional studies to determine which of the identified processes/pathways are directly PNP-dependent and those which are indirectly influenced by PNP.

## Materials and methods

### Arabidopsis cell suspension culture and treatment with AtPNP-A peptide

Cells derived from roots of *A. thaliana* (ecotype Columbia-0) were grown in 100 mL of Gamborg's B-5 (Gamborg et al., 1968) basal salt mixture (Sigma-Aldrich, St. Louis, MO, USA) with 2,4-dichlorophenoxyacetic acid (0.5 μg mL^−1^) and kinetin (0.05 μg mL^−1^) in 250 mL sterile flasks in a growth chamber (Innova® 43, New Brunswick Scientific Co., NJ, USA) at 120 rpm, photosynthetic light set for 12 h light/12 h dark cycles at 23°C, and sub-cultured every 10 days. Cells were treated with biologically active synthetic peptide (GenScript, Piscataway, NJ, USA) containing the active region of AtPNP-A (amino acid 36-69) (Morse et al., 2004; Wang et al., 2007) at the final concentrations of 1 nM and 10 pM or with equal volumes of water as a negative control. Three biological replicates of cells treated with each concentration of peptide or mock-treated cells were collected at 0, 10 and 30 min post-treatment. Media were drained using Stericup® filter units (EMD Millipore, Billerica, MA, USA) and the cells were immediately flash frozen in liquid nitrogen and stored at −140°C until further use (refer to Figure S2).

### Measurement of ROS accumulation after treatment of cells with AtPNP-A

OxiSelect™ Intracellular ROS assay kit (Cell Biolabs Inc., San Diego, CA) was used to measure *in vivo* ROS accumulation as described elsewhere (Marondedze et al., 2013). Ten days after sub-culturing, suspension cultures *A. thaliana* (Col-0) cells were washed three times with Hank's Balanced Salt Solution (HBSS). Cells were plated on 96-well flat-bottom black plate (Greiner Bio-One GmbH, Germany) and incubated for 2 h in a shaking incubator (120 rpm, 23°C). After that the solution was aspirated and discarded, and the cells were then incubated in dark for 1 h at 23°C with 100 μM fluorogenic probe 2′,7′-dichlorodihydrofluorescein diacetate (DCFH-DA) in a total volume of 100 μL. Upon three steps of washing, basal fluorescence of the dye-loaded cells was measured at excitation wavelength of 480 nm and emission at 530 nm using a PHERAstar FS microplate reader (BMG Labtech GmbH, Germany). The cells were then treated with different concentrations of the biologically active AtPNP-A peptide or water (negative control) in a total volume of 100 μL, and the green fluorescence was measured at 10 and 30 min post-treatment as described above. Data from three biological replicates was analyzed using Two-Way ANOVA followed by Tukey's post-test (*p*-value < 0.05 was considered significant).

### Total soluble protein extraction

Approximately 1 g of cells was homogenized for 4 sec twice in 10 volumes of ice-cold 10% (w/v) trichloroacetic acid in acetone using a PowerGen 125 grinder (Fisher Scientific, Rockford, IL, USA), vortexed for 2 min and incubated overnight at −20°C. Precipitated proteins were pelleted by centrifugation using the Allegra® X-22R centrifuge (Beckman Coulter Corp., Brea, CA, USA) at 3,901 × *g* for 20 min at 4°C. The pellet was washed four times with 80% (v/v) ice-cold acetone with vigorous vortexing and subjected to centrifugation at 3,901 × *g* for 20 min at 4°C after each wash. Excess acetone was evaporated by air-drying, and proteins were re-suspended in two volumes of urea lysis buffer [7 M urea, 2 M thiourea, phosphatase inhibitor cocktail set II (Calbiochem, Temecula, CA, USA)] with vigorous vortexing for 3 h at room temperature. The samples were cleared by centrifugation at 3901 × *g* for 20 min at room temperature and total soluble protein concentration was estimated by Bradford assay (Bradford, 1976) using the Quick Start™ Bradford reagent (Bio-Rad, Hercules, CA, USA) and bovine serum albumin as a standard. Aliquots of protein extracts were stored at −80°C until further use.

### Trypsin digestion of extracted proteins

Approximately 1 mg of total soluble protein extract was reduced with 5 mM dithiothreitol for 2 h at 37°C, cooled and then proteins were alkylated with 14 mM iodoacetamide for 30 min at room temperature in the dark. Unreacted iodoacetamide was quenched by increasing dithiothreitol concentration to 10 mM with a further incubation for 15 min at room temperature in the dark. Proteins were diluted to 1.5 M urea with 50 mM triethylammonium bicarbonate (TEAB) buffer (Sigma-Aldrich) and incubated at 50:1 ratio with sequencing-grade modified trypsin (Promega, Madison, WI, USA) overnight at 37°C with gentle agitation. Protein digestion was stopped by acidification of the mixture to pH 2.0 with trifluoroacetic acid. Acidification serves to precipitate lipids that would interfere with downstream purification and importantly to prepare the samples for desalting which requires peptide mixture to be acidic (Hsu et al., 2009). Peptides were then purified using Sep-Pak Vac tC18 100 mg cartridge (Waters, Milford, MA, USA), as described previously (Groen et al., 2013), and completely dried in a Speed Vac concentrator (Thermo Scientific, Bremen, Germany).

### Peptide labeling using tandem mass tag (TMT)

Dried desalted tryptic peptides obtained from the digestion of 1 mg protein were re-suspended in 20% (v/v) acetonitrile and half of the volume was subjected to labeling reaction using tandem mass tag (TMT) sixplex™ isobaric mass tagging kit (Thermo Scientific) performed according to the manufacturer's instructions. Each of the labeling reaction mixtures contained the TMT reagents (0.8 mg) dissolved in 41 μl of anhydrous acetonitrile and approximately 0.5 mg of the tryptic peptides in 95 μL of 20% (v/v) acetonitrile. Each biological replicate of both 1 nM and 10 pM AtPNP-A treated samples were labeled separately with the respective mock treated (water) samples and analyzed independently. Aliquots of the negative control (samples collected at 0, 10, and 30 min) tryptic digests were derivatized with sixplex chemical labels (mock-treated cells collected at 0 min post-treatment with m/z 126 TMT, mock-treated cells collected at 10 min post-treatment with m/z 127 TMT, mock-treated cells collected at 30 min post-treatment with m/z 128 TMT) while tryptic digests of the AtPNP-A treated cells were also derivatized with sixplex chemical labels (AtPNP-A-treated cells collected at 10 min post-treatment with m/z 129 TMT and AtPNP-A-treated cells collected at 30 min post-treatment with m/z 130 TMT). After 1 h incubation, reactions were quenched by 15 min incubation with 8 μl of 5% hydroxylamine. The five labeled samples for each biological replicate in each treatment with either 1 nM or 10 pM AtPNP-A were subsequently combined at equal amounts of 0.5 mg peptides in a total reaction volume of 144 μL per sample and stored at −80°C until further use (refer to Figure S2).

### Peptide fractionation by OFFGEL fractionator

Peptides were fractionated using the 3100 OFFGEL fractionator (Agilent Technologies, Santa Clara, CA, USA) with a 24-well high-resolution immobilized pH gradient strip. Peptide samples were diluted to a final volume of 1.8 mL with 1.25 × peptide OFFGEL stock solution [50% (v/v) glycerol solution, 10% (v/v) OFFGEL buffer pH range 3–10]. Strips were rehydrated, as recommended by the manufacturer, with 40 μL of 1 × immobilized pH gradient strip rehydration solution per well for 15 min and then 150 μL of sample was pipetted into each well. Electrofocusing was carried out to 64 kVh at 20°C, allowing a maximum of 4500 V and 50 μA per strip. After focusing, fractions were separately collected and the wells rinsed twice with 200 μL of a solution containing 50% (v/v) acetonitrile and 5% (v/v) formic acid for 15 min each time. Rinsing solution collected from each well was combined into the tube containing its corresponding fraction. Fractions were dried in a Speed Vac concentrator (Thermo Scientific), re-suspended in 0.1% (v/v) trifluoroacetic acid and purified with Sep-Pak Vac tC18 cartridge (Waters), as previously described (Groen et al., 2013). From each 1 mL fraction of elution per OFFGEL fraction, a volume of 100 μL was subjected to drying in Speed Vac concentrator, suspended in 10 μL of 0.1% (v/v) trifluoroacetic acid and further purified using ZipTipC_18_ (P-10) pipette tips (EMD Millipore) according to manufacturer's recommendations. Peptides eluted with solution containing 0.1% (v/v) trifluoroacetic acid in 50% (v/v) acetonitrile were dried in Speed Vac concentrator in preparation for mass spectrometric analysis.

### Protein identification by LTQ orbitrap

Dried peptides were re-suspended in 5% (v/v) acetonitrile and 0.1% (v/v) formic acid and analyzed by an LTQ Orbitrap Velos™ mass spectrometer (Thermo Scientific) operated as described previously (Groen et al., 2013) in positive mode and coupled with a nanoelectrospray ion source (Proxeon Biosystems, Odense, Denmark) for nano-LC-MS/MS analyzes. Samples obtained from each biological replicate of 1 nM AtPNP-A-treated cells were analyzed on LC-MS/MS in technical triplicates as was the first biological replicate of 10 pM peptide-treated cells. After running three technical replicates on all biological replicates of the first treatment (1 nM AtPNP-A), we found the third replicate did not increase the overall identification of peptides and as a result we resorted to two technical replicates, which was also cost effective, for the final two biological replicates of 10 pM AtPNP-A-treated cells. A volume of 5 μL of peptide mixtures was injected onto a Magic C18AQ 5 μm, 200 Å, 0.3 mm × 50 mm long pre-column (Michrom, Auburn, CA, USA) and a Magic C18AQ 3 μm, 200 Å, 0.1 mm × 150 mm long column (Michrom), and a spray voltage of 1500 V was applied. The mobile phases consisted of 0.1% (v/v) formic acid and 5% (v/v) acetonitrile (phase A) and 0.1% (v/v) formic acid and 90% (v/v) acetonitrile (phase B). A three-step gradient of 0–40% phase B in 20 min, then 40–90% phase B in 5 min, and finally 90% phase B for 20 min with a flow of 300 nL min^−1^ over 45 min was applied for peptide elution. The MS scan range was *m/z* 350–1600 and the normalized collision-induced dissociation at 35.0 V. The top 10 precursor ions were selected in the MS scan by Orbitrap with resolution *r* = 60.000 for fragmentation in the linear ion trap. The spray voltage was set at 1.5 kV, the capillary voltage 47.5 V, the capillary temperature 250°C, and the sheath and auxiliary gas flow at 35 and 15, respectively. Data were recorded with the Xcalibur software version 2.1 (Thermo Scientific) and converted from “.raw” to “.mgf” with Proteome Discover version 1.2.0.208 (Thermo Scientific). All spectra were submitted to a local MASCOT (Matrix Science, London, UK) and SEQUEST (Thermo Scientific) servers and searched against *A. thaliana* in the TAIR database (release 10), with a precursor mass tolerance of 10 ppm, a fragment ion mass tolerance of ±0.5 Da, and strict trypsin specificity allowing up to one missed cleavage, carbamidomethyl modification on cysteine residues as fixed modification, and oxidation of methionine residues and phosphorylation of serine, threonine and tyrosine residues as variable modifications. SEQUEST and MASCOT were used only to identify proteins from the LC/MSMS spectra which were then all evaluated and quantified using Scaffold Q+ software detailed below (see Figures S2, S3).

### Quantification of differentially expressed proteins

Quantitative analysis of the protein expression levels, determined by tandem mass spectrometry of TMT-labeled peptides, was performed with Scaffold Q+ software, version 4.0.4 (Proteome Software, Portland, USA). The Scaffold software validates peptides from various database search engines including Mascot by using PeptideProphet algorithm (Searle, 2010). The algorithm is an alternative to the threshold model that converts scores and penalties used in different search engines into a single discriminant score so the agreement between search engines is calculated and factored into a combined peptide probability. The proteins were normalized on the basis of the assumption that total intensity remained the same for each of the tags used and we used a false discovery rate (FDR) of 0.7%. Expression levels of proteins that were considered positive identifications from AtPNP-A-treated cells (in at least one technical replicate) were compared with mock-treated cells collected at the same time-point. Differential expression of a protein was considered significant if the fold change of a quantity of the protein estimated upon combining data from technical replicates corresponding to the peptide-treated and mock-treated cells for a given biological replicate collected at the same time-point was greater or equal to |±1.5|, verified by Mann–Whitney test (*p*-value < 0.05), in at least two out of three biological replicates. Proteins can be identified by multiple peptides spectral counts however some of the peptide sequences may be shared amongst several proteins. We chose to use only unique peptide spectra, not those of redundant peptides which may reflect the presence of a different protein. Thus, proteins were considered positive identifications if they were identified with a minimum of one unique peptide (SEQUEST Xcorr > 2 or MASCOT ion score > 32 and a peptide probability of 90%) at the protein threshold of 95% (refer to Figures S2, S3).

### Gene ontology (GO) and gene expression analyzes

The gene ontology (GO) and functional categorization analyzes of proteins considered differentially expressed were performed using TAIR GO search (http://www.arabidopsis.org/tools/bulk/go/index.jsp; October 2014). Transcriptional profiles of proteins that are affected by AtPNP-A were analyzed using Genevestigator [https://www.genevestigator.com/gv/plant.jsp (Zimmermann et al., 2004); February 2014].

## Results

### AtPNP-A induces ROS production

**Figure 2:**
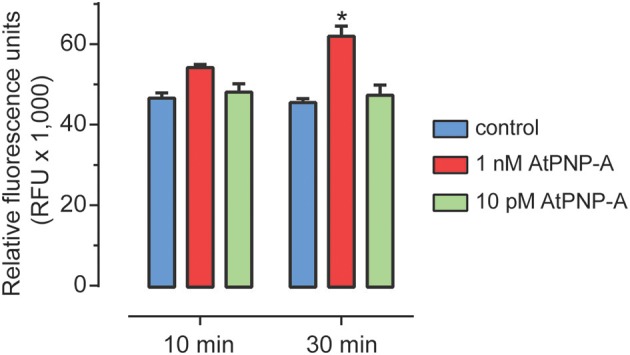
**ROS accumulation following AtPNP-A treatment**. ROS accumulation after treatment of suspension-cultured cells of *A. thaliana* (Col-0) with AtPNP-A. Relative fluorescence was measured at 10 and 30 min post-treatment. Data are mean ± s.e.m. of three biological replicates (*n* = 3). The bar with an asterisk (*) indicates data that is significantly different (*p*-value < 0.05; Two-Way ANOVA, Tukey post-test).

Previous studies have shown that AtPNP-A triggers a number of physiological responses in whole plants, tissue and cell suspension cultures. AtPNP-A is mainly produced in leaf mesophyll cells and to a lesser extent in cortical cells in stems and petioles (Wang et al., 2011). No AtPNP-A promoter:GUS expression was detected in roots (Wang et al., 2011). Therefore, to avoid confounding effects of endogenous production, we used *A. thaliana* (Col-0) suspension cells derived from root cells. We could not reliably detect any AtPNP-A specific peptide products in these cells under our experimental conditions. The effect of the biologically active peptide corresponding to residues 36–69 of AtPNP-A (Figure S1) on ROS production was examined. At 1 nM AtPNP-A peptide, ROS production was significantly induced within 30 min in suspension-cultured *A. thaliana* (Col-0) cells (Figure 2). Concentrations equal to or lower than 10 pM did not induce any significant changes in ROS production at the time points used.

### Examination of the AtPNP-A induced proteome

In order to obtain insight into the proteins that are differentially expressed in response to AtPNP-A, we followed the response of suspension-cultured *A. thaliana* (Col-0) cells to physiological concentrations of biologically active plant peptide at two different time points post-treatment. To reveal the quantitative and temporal relationships between the concentrations of AtPNP-A used and corresponding protein level, TMT sixplex was applied since it allows up to six different samples to be compared in a single mass spectrometry run, thus diminishing variations introduced during sample preparation and spectrum MS acquisition. Preliminary experiments indicated that peptide corresponding to the active site of AtPNP-A elicits alterations in the protein production of suspension-cultured *A. thaliana* (Col-0) cells not only in nano- but also at lower concentrations. Since 1 nM was the lowest concentration of AtPNP-A that led to statistically significant ROS modulation (Figure 2), while application of 100-fold lower concentration of the peptide did not give significant response at 10 or 30 min post-treatment, we aimed to identify whether protein expression is differentially regulated during the ROS-dependent and ROS-independent signaling events triggered by AtPNP-A. Consequently, 1 nM and 10 pM concentrations of AtPNP-A and the sampling time-points of 10 and 30 min post-treatment were chosen to obtain an overview of protein expression differentially regulated by AtPNP-A in a concentration- and time-dependent manner.

Samples from three biological replicates were collected at 0, 10, and 30 min after treatment. Equal amounts of soluble proteins for each sample were enzymatically digested and tryptic peptides were then labeled with one of the five isobaric TMTs used. Tagged samples were combined and subjected to LC-MS/MS analysis and quantitative analysis of identified proteins was performed for the biological triplicates for each concentration of AtPNP-A. All proteins identified at each time point upon treatment with each concentration were subjected to quantitative analyzes (so that proteins identified 10 min and 30 min upon treatment with the same concentration of AtPNP-A were analyzed separately). A total of 4641 proteins in the case of cellular response to 1 nM AtPNP-A and a total of 3447 proteins in the case of cellular response to 10 pM AtPNP-A was revealed at FDR of 0.7%. Only a few of these proteins fulfilled our requirement [at least |±1.5| fold-change (Mann–Whitney test, *p*-value < 0.05) per each biological replicate and in at least 2 out of 3 biological replicates (per each time point and peptide concentration used)] for being considered as differentially expressed upon treatment with AtPNP-A with good confidence. Details of all differentially expressed proteins can be found in the Tables S1, S2. The first group includes 11 proteins differentially expressed in response to 1 nM AtPNP-A (Table 1), while the second group yielded 15 proteins differentially expressed in response to 10 pM AtPNP-A (Table 2).

**Table 1:** **Annotation of proteins differentially expressed 10 and 30 min after treatment of cells with 1 nM AtPNP-A peptide**.

**AGI ID**	**Protein annotation**	***p*-value**	**Log_2_ fold change**	**GO term (BP)**
**PROTEINS WITH EXPRESSION DIFFERENTIALLY REGULATED 10 MIN POST-TREATMENT**				
AT2G37230.1	Tetratricopeptide repeat (TPR)-like superfamily protein	0.0001	1.10	
AT1G52300.1	Zinc-binding ribosomal protein family protein (RPL37B)	0.0001	1.05	A
AT2G32120.1	Heat-shock protein 70T-2 (HSP70T-2)	0.0001	0.95	
AT5G07470.1	Peptidemethionine sulfoxide reductase 3 (PMSR3)	0.0001	0.95	B, C
AT3G58640.1	Mitogen activated protein kinase kinase kinase-related	0.0210	0.75	
AT3G29090.1	Pectin methylesterase 31 (PME31)	0.0001	0.70	
AT3G16410.1	Nitrile specifier protein 4 (NSP4)	0.0001	−0.80	
**PROTEINS WITH EXPRESSION DIFFERENTIALLY REGULATED 30 MIN POST-TREATMENT**				
AT1G52300.1	Zinc-binding ribosomal protein family protein (RPL37B)	0.0001	1.00	A
AT4G23670.1	Polyketide cyclase/dehydrase and lipid transport superfamily protein	0.0001	0.85	D
AT5G17820.1	Peroxidase superfamily protein (Prx57)	0.0001	0.65	C, E
AT2G37970.1	SOUL heme-binding family protein (SOUL-1)	0.0008	−0.70	
AT2G41730.1	Unknown protein	0.0002	−0.80	

*Only proteins showing significant (p-value < 0.05; Mann–Whitney test) differential expression, with at least 1.5-fold change in at least 2 out of 3 biological replicates, are presented. Gene Ontology (GO) terms include biological process (BP) category. A – Translation (GO:0006412). B – Cellular membrane fusion (GO:0006944); C – Oxidation-reduction process (GO:0055114); D – Response to salt stress (GO:0009651); E – Root hair elongation (GO:0048767); AGI, Arabidopsis Genome Initiative*.

**Table 2:** **Annotation of proteins differentially expressed 10 and 30 min after treatment of cells with 10 pM AtPNP-A peptide**.

**AGI ID**	**Protein annotation**	***p*-value**	**Log_2_ fold change**	**GO term (BP)**
**PROTEINS WITH EXPRESSION DIFFERENTIALLY REGULATED 10 MIN POST-TREATMENT**				
AT5G08040.1	Mitochondrial import receptor subunit TOM5 homolog (TOM5)	0.0000	1.40	
AT3G55010.1	Phosphoribosyl-aminoimidazole synthetase (PUR5)	0.0001	0.85	F
AT1G54410.1	Dehydrin family protein (HIRD11)	0.0001	0.80	
AT1G17880.1	Basic transcription factor 3 (BTF3)	0.0000	0.70	D
AT1G09795.1	ATP phosphoribosyl transferase 2 (ATP-PRT2)	0.0000	−0.80	
AT5G14340.1	Myb domain protein 40 (MYB40)	0.0000	−0.83	
AT4G14430.1	Indole-3-butyric acid response 10 (IBR10)	0.0000	−0.93	E, G
AT1G07660.1	Histone superfamily protein	0.0001	−0.93	
**PROTEINS WITH EXPRESSION DIFFERENTIALLY REGULATED 30 MIN POST-TREATMENT**				
AT1G78150.1	Unknown protein	0.0027	0.75	
AT3G49601.1	Unknown protein	0.0071	0.65	
AT3G04184.1	Unknown protein	0.0280	−0.63	
AT5G41520.1	RNA binding Plectin/S10 domain-containing protein	0.0090	−0.64	A
AT4G23895.3	Pleckstrin homology (PH) domain-containing protein	0.0004	−0.70	
AT3G28710.1	ATPase, V0/A0 complex, subunit C/D	0.0041	−0.86	
AT3G62250.1	Ubiquitin 5 (UBQ5)	0.0008	−1.15	A

*Only proteins showing significant (p-value < 0.05; Mann–Whitney test) differential expression, with at least 1.5-fold change in at least 2 out of 3 biological replicates, are presented. Gene Ontology (GO) terms include biological process (BP) category. A – Translation (GO:0006412). D – Response to salt stress (GO:0009651); E – Root hair elongation (GO:0048767); F – Nucleotide biosynthetic process (GO:0009165); G – Response to water deprivation (GO:0009414); AGI, Arabidopsis Genome Initiative*.

### Proteins differentially expressed upon treatment with AtPNP-A

Proteins differentially expressed 10 and 30 min after treatment with 1 nM AtPNP-A are given in Table 1. Only one of the proteins up-regulated at 10 min is sill up-regulated 30 min after 1 nM AtPNP_A treatment and this protein participates in “translation” (Table 1). This may indicate AtPNP-A dependent enhancement of intracellular changes at transcriptional and/or translational level known to occur in cells upon treatment with AtPNP-A (Meier et al., 2008; Wang et al., 2011). Another protein differentially upregulated after 30 min of AtPNP-A treatment is annotated to be “upregulated in salt stress.” Interestingly, two proteins with up-regulated expression at either 10 or 30 min post-treatment are reported to have a role in “oxidation-reduction processes” (Table 1), confirming that AtPNP-A exerts some of its functions—either directly or indirectly—*via* a modulation of the cellular redox state (Figure 2).

The proteins showing up- or down-regulation of their expression 10 or 30 min after treatment with 10 pM AtPNP-A are listed in Table 2. Differentially expressed proteins following 10 pM AtPNP-A treatment included the GO terms: “response to salt stress,” “nucleotide biosynthetic process,” “response to water deprivation,” and “root hair elongation” (Table 2). The function of several of the proteins positively-regulated after the treatment is unknown, while forty percent of proteins down-regulated at 30 min upon treatment with 10 pM AtPNP-A are involved in “translation.”

**Figure 3:**
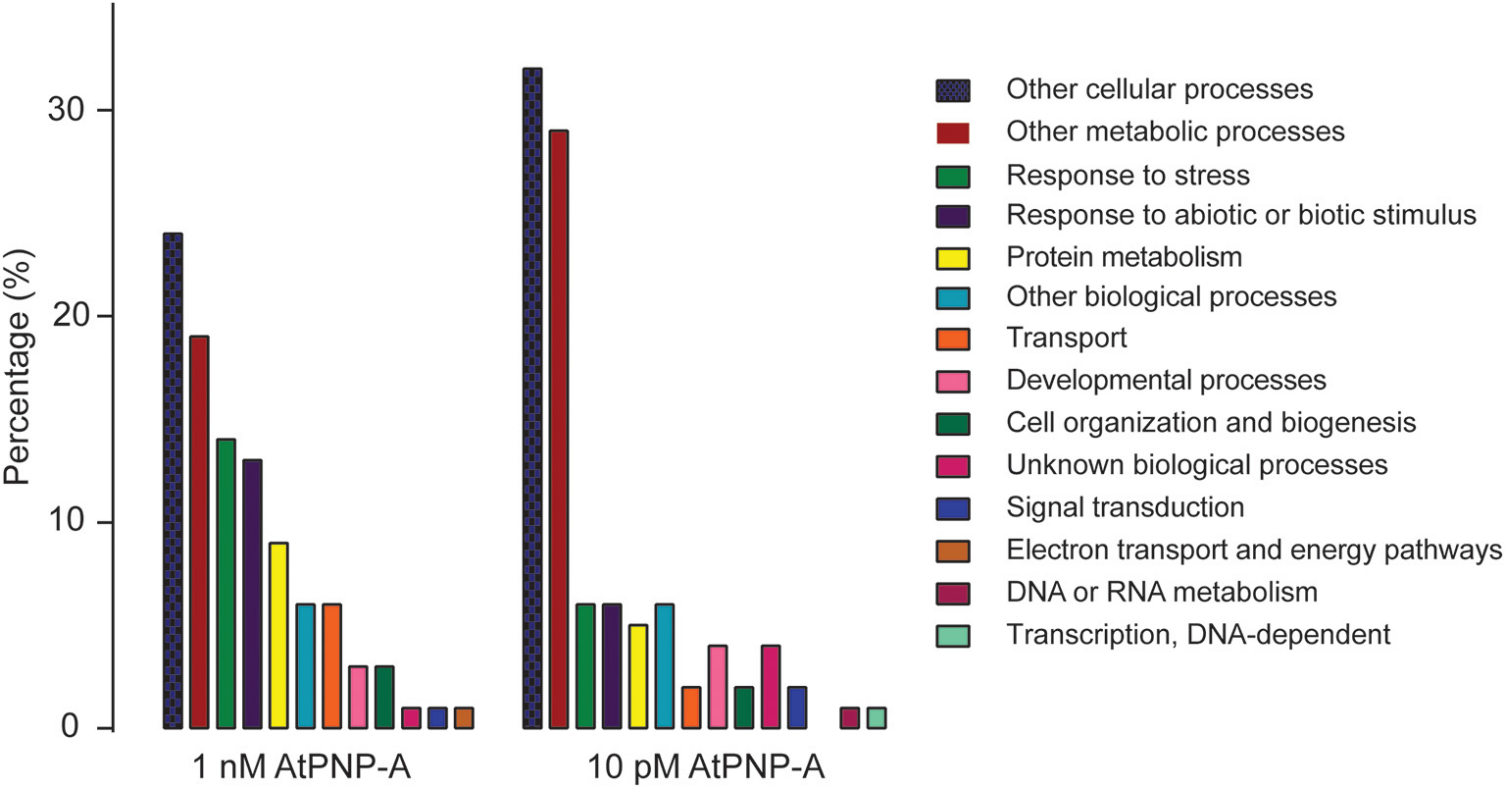
**Comparison of GO categories differentially regulated by AtPNP-A at 1 nM and 10 pM concentration**.

The cellular responses to AtPNP-A appear to be highly concentration-dependent; the proteins differentially expressed upon treatment with 1 nM AtPNP-A do not overlap with the pool of proteins differentially expressed upon treatment with 10 pM peptide. Nevertheless, the majority of GO terms assigned to the proteins differentially expressed upon treatment with both concentrations of AtPNP-A, either at 10 or 30 min post-treatment, correspond to physiological functions modulated by AtPNP-A in plant systems. For instance, GO terms related to responses to abiotic stress, such as “translation” and “response to salt stress” (Tables 1, 2), are present among proteins showing AtPNP-A-dependent regulation of expression upon treatment with both concentrations of the peptide. When the proportion of differentially annotated GO terms is analyzed, it is evident that there is a concentration dependent increase in the GO terms relating to response to stress (6–14%), response to biotic and abiotic stimuli (6–13%), protein metabolism (5–9%), transport (2–6%), cell organization and biogenesis (2–3%), and electron transport or energy pathways (up to 1%) (Figure 3). On the other hand, representation of functional categories such as other cellular processes (24–32%), other metabolic processes (19–29%), unknown biological processes (1–4%), signal transduction (1–2%), and developmental processes (3–4%) in the pool of proteins differentially expressed upon treatment with 10 pM AtPNP-A (Figure 3) indicates that modulation of expression of proteins characterized by GO terms relating to these biological processes is inversely related to the concentration of the AtPNP-A peptide used.

## Discussion

Plant NPs affect salt and water homeostasis, which is not dissimilar to the functions exerted by their analogs found in vertebrate systems. Increases in osmoticum induce AtPNP-A production (Rafudeen et al., 2003; Wang et al., 2011) and transcriptomics analysis has revealed that *AtPNP-A* is also strongly up-regulated in response to biotic stresses (Meier et al., 2008). Biochemical and physiological responses of plants to treatment with a recombinant protein of AtPNP-A include induction of an increase in stomatal conductance and transpiration rate (Gottig et al., 2008), elevation of leaf dark respiration rate (Ruzvidzo et al., 2011), and enhanced radial water movement out of xylem (Suwastika and Gehring, 1998) observed at micromolar concentrations of the protein, as well as induction of cGMP-dependent stomatal guard cell opening (Morse et al., 2004), modulation of osmoticum-dependent volume changes in leaf mesophyll cell protoplasts exerted by the recombinant protein and peptide corresponding to the amino acid sequence of the active site of the protein (Wang et al., 2007) reported at nanomolar concentrations of the recombinant AtPNP-A protein. In addition, AtPNP-A modulates ion flux in the root in a developmental stage-specific and tissue-specific manner (Ludidi et al., 2004). Although the importance of PNPs in maintaining ion and fluid balance in response to abiotic and biotic stress has been established (Figure 1), the molecular mode of their action remains largely unknown.

In order to shed light on the AtPNP-A-dependent response at the systems level changes in protein profiles have been obtained and analyzed. To explore the physiologically relevant concentration effect of peptides corresponding to the active site of AtPNP-A on the proteome, 1 nM and 10 pM concentrations were chosen and tested on root-derived cells in suspension cultures. It is possible that shaking conditions induce AtPNP-A production but AtPNP-A specific peptides were not detectable in either the mock treated control or synthetic peptide treated cell suspension cultures indicating that endogenous production of AtPNP-A was below the detectable levels. Application of these two concentrations enabled us not only to pinpoint sets of proteins where expression is differentially regulated by AtPNP-A, but it also allowed for differentiation of AtPNP-A-dependent signaling events that involve or do not involve ROS modulation (Figure 2). Although nanomolar concentrations of AtPNP-A give significant physiological effects (Maryani et al., 2001; Wang et al., 2007; Gottig et al., 2008) (Figure 2), the differential regulation of protein expression upon application of 10 pM peptide indicates that picomolar concentrations of AtPNP-A also elicits cellular responses (Table 2). Representation of GO terms in sets of differentially expressed proteins was used as the basis for inferring cellular responses modulated by AtPNP-A. The functional interpretation of experimental data using the GO domain of biological process provides an overview of AtPNP-A-dependent cellular events.

### Proteins differentially regulated in response to stress

The GO biological process term “response to salt stress” is represented in the group of proteins where expression is regulated by nano- and picomolar concentrations of AtPNP-A. The expression of polyketide cyclase/dehydrase and lipid transport superfamily protein (At4g23670.1, also known as major latex protein-related) is up-regulated 30 min after the treatment with nanomolar AtPNP-A. Transcripts of the polyketide cyclase/dehydrase and lipid transport superfamily protein are transiently up-regulated in Arabidopsis root apices upon gravity stimulation (Kimbrough et al., 2004), in leaves subjected to a combination of drought and heat stress (Rizhsky et al., 2004), and in roots upon exposure to low-level ionizing cesium (^134^Cs) (Sahr et al., 2005). Moreover, the protein appears to play a role in seed dormancy (Chibani et al., 2006) as well as in plant defense since it has been reported to accumulate in response to pathogen-associated molecular patterns (PAMPs) (Jones et al., 2006).

The expression of basic transcription factor (BTF3; At1g17880.1) was up-regulated following application of 10 pM AtPNP-A and is also involved in salt stress (Table 2). BTF3 is located in the nucleus where it interacts with eukaryotic translation initiation factor 4E2 (At5g35620.1) (Freire, 2005), conceivably part of the induction of transcriptional changes in response to AtPNP-A. Transcription level of BTF3 is decreased upon sucrose starvation (Giege et al., 2005), and microarray data revealed a similar pattern of *BTF3* and *AtPNP-A* expression in conditions of abiotic and biotic stress, including cold stress or glucose treatment, exposure to high light and infection with *Golovinomyces cichoracearum*. A quantitative study employing isobaric tags for relative and absolute quantitation revealed pronounced elevation of BTF3 abundance in short-day-grown *cat2* (catalase 2; At4g35090.1) and *nca1* (no catalase activity 1; At3g54360.1) mutants that display loss of sensitivity to bacterial effector avrRpm1 and to hydroxyurea (Hackenberg et al., 2013). This suggests a role of BTF3 in autophagy-dependent programmed cell death.

Other proteins involved in stress or responses to biotic and abiotic stimuli are also up-regulated by AtPNP-A treatments. Expression of heat-shock protein 70T-2 (HSP70T-2; At2g32120.1) is enhanced by nanomolar AtPNP-A (Table 1) and is also regulated by wounding and other signals such as elevated 12-oxo-phytodienoic acid, jasmonic acid and strongly by wounding (Taki et al., 2005). Pectin methylesterase 31 (PME31; At3g29090.1) is also raised by 1 nM AtPNP-A (Table 1) and is a partly characterized cytosolic protein (Dedeurwaerder et al., 2009) that is a member of an extensive family of proteins that de-methylesterify pectin. Along with several other members of the PME family, PME31 is activated in response to *Pseudomonas syringae* attack where it contributes to immune response in a jasmonic acid dependent process (Bethke et al., 2014). The differential regulation of these proteins is in agreement with the earlier findings involving the interaction of AtPNP-A and pathogen PNP-A-like molecules in host-pathogen interactions (Gottig et al., 2008; Meier et al., 2008; de Jonge et al., 2012).

### Proteins involved in oxidation-reduction process

Two proteins with a role in oxidation-reduction process are up-regulated in response to nanomolar amounts of AtPNP-A (Table 1) and one protein following picomolar treatments. The methionine sulfoxide reductase 3 (PMSR3; At5g07470.1), which is also involved in cellular membrane fusion, is differentially regulated during the early-stage (10 min) response to 1 nM AtPNP-A. Expression of peroxidase superfamily protein (Prx57; At5g17820.1) is up-regulated 30 min post-treatment. AtPNP-A-dependent PMSR3 expression may occur as an early-stage response of the cells to oxidative stress (Figure 2) and is a direct indication of a role in the maintenance of the redox balance. PMSR3 belongs to a family of ubiquitously expressed enzymes responsible for the repair of oxidation-damaged proteins. PMSR3 catalyzes the thioredoxin-dependent reduction of methionine sulfoxide to methionine (Brot et al., 1981) as well as reducing free methionine sulfoxide residues (Grimaud et al., 2001). Generation of methionine sulfoxide is caused by biological oxidants and metals and can lead to conformational modifications and changed activity of proteins. PMSR3 is a cytosolic isoform within the five-gene PMSR subfamily A in *A. thaliana*, and is expressed in roots and stems (Rouhier et al., 2006). Since overexpression of PMSR3 has been reported to increase phosphorylation of Arabidopsis nitrate reductase in normal leaves in the dark (Hardin et al., 2009), AtPNP-A dependent accumulation of PMSR3 may have a significant impact on phosphorylation events occurring in the cells and thereby affect plant redox homeostasis. AtPNP-A causes a rapid increases in cGMP (Pharmawati et al., 1998a, 2001; Morse et al., 2004; Wang et al., 2007) and cGMP induces specific methionine oxidation of many stress-response proteins in *A. thaliana* (Marondedze et al., 2013) and significantly affects H_2_O_2_-dependent K^+^ and Na^+^ net-fluxes in Arabidopsis roots (Ordoñez et al., 2014). Taken together, these observations are consistent with a role of AtPNP-A in cGMP-dependent redox signaling and responses.

Given the ubiquitous presence of PMSRs, we speculate that altered physiological performance of the rough lemon (*Citrus jambhiri*) infected with the bacterial citrus pathogen *Xanthomonas axanopodis* pv. c*itri* expressing XacPNP, a PNP-like molecule (Nembaware et al., 2004), may to some extend result from up-regulation of plant MSR(s) expression. Similarly to AtPNP-A, XacPNP also has the ability to induce stomatal opening and cGMP-dependent protoplast swelling that is strongly inhibited in the presence of cycloheximide (Gottig et al., 2008), but has also been reported to be responsible for altering host photosynthesis and the formation of wet lesions (Garavaglia et al., 2010a). Up-regulation of *PMSR3* transcripts is also seen in transcriptomics studies in response to attack by different pathogens and supports this hypothesis.

*Prx57* encodes a protein involved in the oxidation-reduction process and is up-regulated 30 min after treatment with AtPNP-A. Transcriptomic analysis of this gene indicates that it is down-regulated under conditions of oxidative stress caused by drought or cold stress (Genevestigator). The expression of *Prx57* has been demonstrated as negatively regulated by ethylene (De Paepe et al., 2004) and positively-regulated by arsenate stress (Abercrombie et al., 2008). Prx57 belongs to class III peroxidases (Welinder et al., 2002), which have been implicated in variety of functions including cell elongation, cell wall differentiation and defense against pathogens and are located in vacuoles and cell walls (Passardi et al., 2005). Apoplastic localization enables them to directly modify cell wall structure, either by causing cross-linking or cleaving of the cell wall polysaccharides, leading to either stimulation or restriction of cell growth. A recent study has revealed that MYB-like transcription factor (At5g47390.1) is a negative regulator of *Prx57* expression and peroxidase activity (Lu et al., 2014). Moreover, an overexpression line for *Prx57* showed significant reduction of both leaf and cell size, but did not affect cell number, compared with the wild-type plants. These observations may implicate AtPNP-A in cell wall re-modeling during growth. It is conceivable that AtPNP-A-dependent up-regulation of Prx57 expression may result in increased cell wall rigidity which in turn strengthens the physical barrier that limits pathogen invasion.

The dehydrin family protein (HIRD11; At1g54410.1) is up-regulated in response to 10 min treatment with 10 pM AtPNP-A (Table 2). HIRD11 is a KS-type dehydrin that inhibits production of hydrogen peroxide and hydroxyl radicals in the Cu-ascorbate system (Hara et al., 2013). Thus, expression and activation HIRD11 can result in the suppression of ROS formation and this could explain why no ROS response was detected at 10 pM AtPNP-A. However, over-representation of proteins involved in oxidation-reduction process in the set of proteins differentially expressed upon treatment with nanomolar (Table 1) concentrations of AtPNP-A is in agreement with the results of ROS accumulation (Figure 2). Together these findings confirm that the components of the AtPNP-A signaling cascade are tightly regulated by the concentration of the peptide applied.

Several physiological responses to AtPNP-A have been demonstrated as significantly reduced when protein synthesis is inhibited by cycloheximide (Rafudeen et al., 2003; Gottig et al., 2008) and this implied that some responses to AtPNP-A require *de novo* protein synthesis. Responses to stress events involve changes in protein expression and thus it is not surprising that some of the proteins modulated by AtPNP-A treatment are involved in translation (Tables 1, 2).

In summary, distinct AtPNP-A dependent changes of the proteome signature occur at nanomolar and picomolar concentrations, while only the higher concentration causes ROS accumulation. We therefore conclude that firstly, our treatment concentrations are in the right range and secondly, that the induction of some proteins may be caused by an indirect ROS-dependent effect and this effect could be considered a type of cellular priming to ensure responsiveness to higher and more physiologically relevant PNP concentrations. We propose that AtPNP-A, possibly signaling through cGMP, has a key role in oxidation-reduction processes as well as response to salt stress.

## Author contributions

Ilona Turek and Chris Gehring designed the work, Ilona Turek and Claudius Marondedze undertook the work. All authors were involved in the analysis and interpretation of the work and drafting, revising and approving the manuscript.

### Conflict of interest statement

The authors declare that the research was conducted in the absence of any commercial or financial relationships that could be construed as a potential conflict of interest.

## Supplementary material

Figure S1 **Overview of PNP-A domains**.

Figure S2 **Overview of TMT experiment**.

Figure S3 **Overview of MS data analysis**.

Table S1 **1 nM AtPNP treatment**.

Table S2 **10 pM AtPNP treatment**.

Table S3 **Key to 1 nM AtPNP-A treatment spectra**.

Table S4 **Key to 10 pM AtPNP-A treatment spectra**.

The mass spectrometry proteomics data have been deposited to the ProteomeXchange Consortium (Vizcaíno et al., 2014) via the PRIDE partner repository with the dataset identifier PXD001386 and DOI: 10.6019/PXD001386. See Tables S3, S4 for a key to the data.

## Abbreviations

ANP: atrial natriuretic peptide
BP: biological processes
BTF3: basic transcription factor 3
GO: gene ontology
HIRDII: dehydrin family protein
HSP70T: heat-shock protein 70T-2
PME31: pectin methylesterase 31
PMSR3: peptidemethionine sulfoxide reductase 3
PNP: plant natriuretic peptide
Prx57: peroxidase superfamily protein
ROS: reactive oxygen species
TMT: tandem mass tag.
