# Supplementary material for: Plant natriuretic peptides induce proteins diagnostic for an adaptive response to stress
Source: Front Plant Sci. 2014 Nov 26;5:661. doi: 10.3389/fpls.2014.00661 (PMC4244590; doi:10.3389/fpls.2014.00661)
Supplement: Figure S1 — Overview of PNP-A domains. [file Image1.PDF]

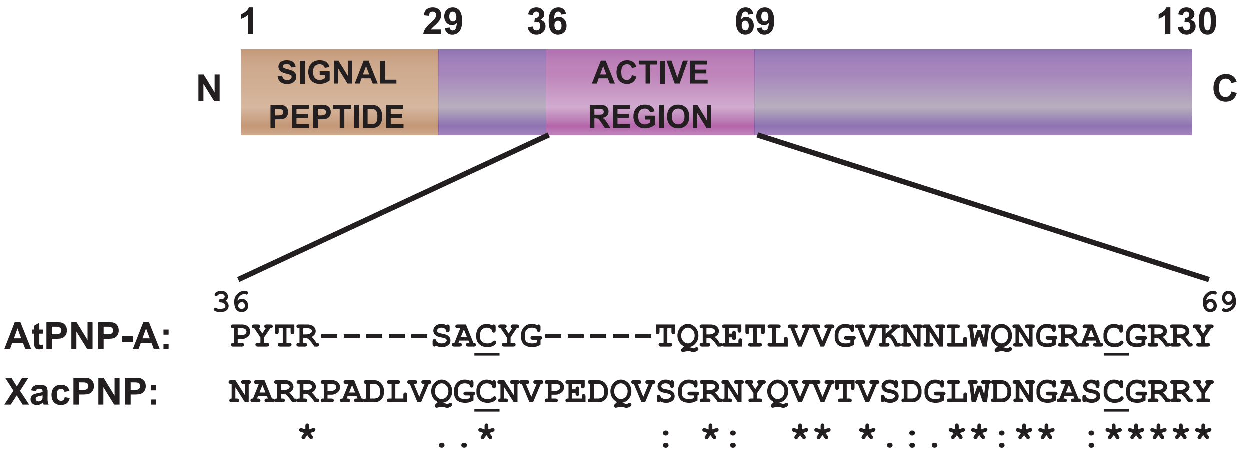

Supplementary Figure 1. Overview of PNP-A domains.
AtPNP-A contains a signal peptide and an active region found closer to the N-terminal of the protein. The homology between the active region of AtPNP-A and XacPNP is shown.
