# Supplementary material for: Plant natriuretic peptides induce proteins diagnostic for an adaptive response to stress
Source: Front Plant Sci. 2014 Nov 26;5:661. doi: 10.3389/fpls.2014.00661 (PMC4244590; doi:10.3389/fpls.2014.00661)
Supplement: Figure S2 — Overview of TMT experiment. [file Image2.PDF]

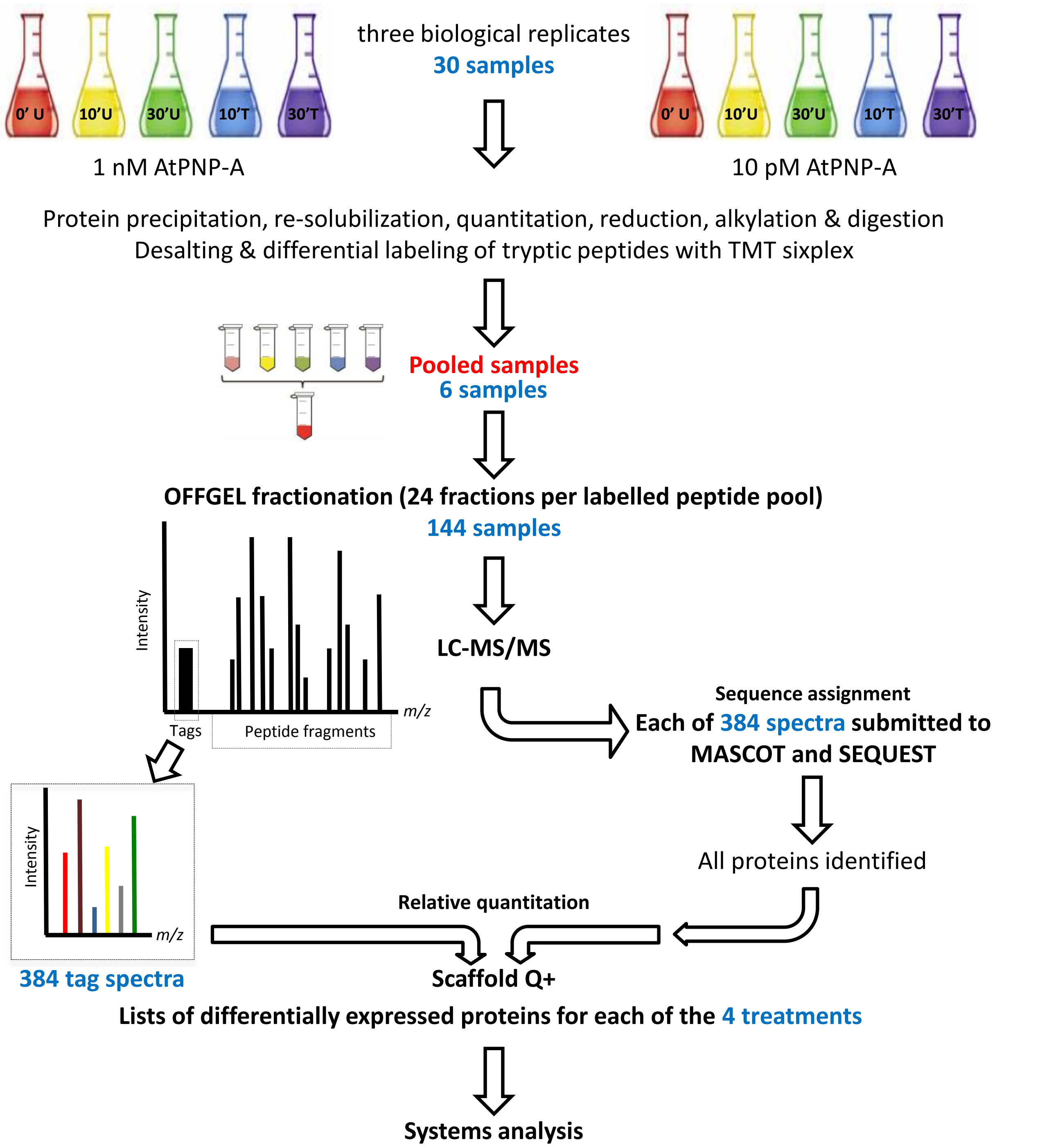

Supplementary Figure 2. Overview of TMT experiment.
Arabidopsis suspension cells were treated with AtPNP-A (1nM or 10 pM) or water (mock) for 0, 10 or 30 min to establish the 4 treatments (10 min 1 nM AtPNP-A, 30 min 1 nM AtPNP-A, 10 min 10 pM AtPNP-A and 30 min 10 pM AtPNP-A) and corresponding mock treatments. Three biological replicates were performed for each resulting in 30 samples. Each sample then independently underwent protein precipitation, re-solubilization, quantitation, reduction, alkylation and digestion followed by desalting before differential labeling of tryptic peptides with TMT sixplex. AtPNP-A-treated cells collected at 10 and 30 min post-treatment were labelled with m/z 129 TMT and m/z 130 TMT while the 0, 10, 30 min mock treated cells were labelled with m/z 126 TMT, m/z 127 TMT and m/z 128 TMT, respectively. Equal amounts of labelled protein from the corresponding biological replicates were then pooled to create 6 pooled samples (3 biological replicates for 1 nM AtPNP-A treatments and 3 biological replicates for 10 pM AtPNP-A treatments). OFFGEL fractionation gave 24 fractions per labelled peptide pool resulting in 144 samples (72 samples 1 nM AtPNP-A treatment, 72 samples 10 pM AtPNP-A treatment). Each of these samples was analyzed by LC/MSMS in duplicate or triplicate resulting in 384 spectra (see Supplementary Tables 3 and 4). Each spectra was analyzed independently by MASCOT and SEQUEST for sequence assignment (see Supplementary Tables 1 and 2). Scaffold Q+ was then used with the tagged MS spectra to relatively quantify all identified proteins. Differential protein expression was considered significant if the combined data from pooled technical replicates for a given biological replicate was greater or equal to $|\pm 1.5|$ of the related mock treatment, verified by Mann-Whitney test (p-value < 0.05), in at least two out of three biological replicates (see Tables 1 and 2 and Supplementary Figure 3 for more detail). Differentially expressed proteins were then examined by gene ontology and gene expression analysis.
