# Supplementary material for: Plant natriuretic peptides induce proteins diagnostic for an adaptive response to stress
Source: Front Plant Sci. 2014 Nov 26;5:661. doi: 10.3389/fpls.2014.00661 (PMC4244590; doi:10.3389/fpls.2014.00661)
Supplement: Figure S3 — Overview of MS data analysis. [file Image3.PDF]

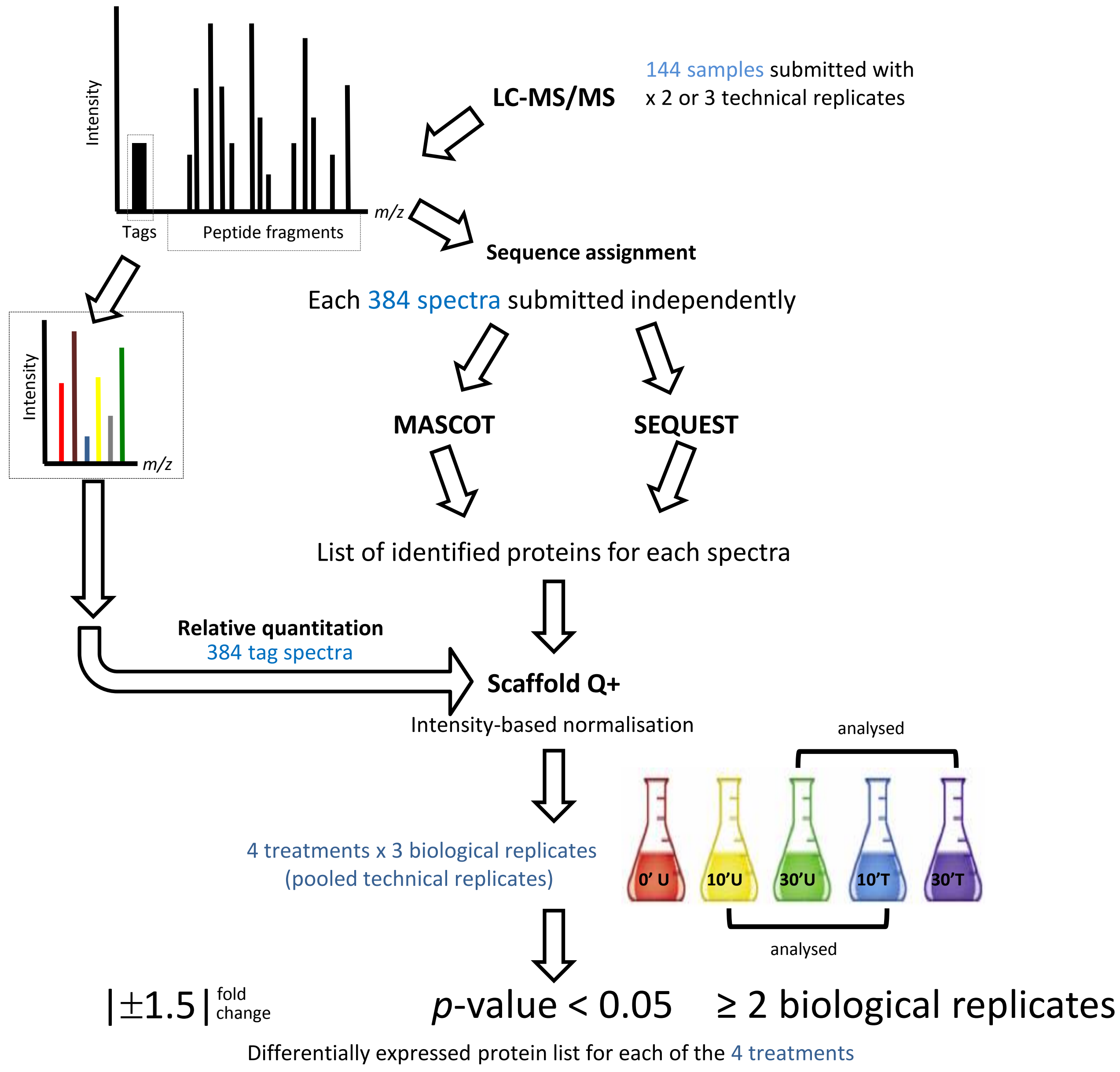

Supplementary Figure 3. Overview of MS data analysis.

144 samples representing the 24 fractions for each of 3 biological replicates of the 4 treatments (10 min 1 nM AtPNP-A, 30 min 1 nM AtPNP-A, 10 min 10 pM AtPNP-A and 30 min 10 pM AtPNP-A) were submitted for MS analyses. Duplicate or triplicate technical replicates for each sample resulted in 384 spectra (see Supplementary Tables 3 and 4). Each MS spectra was independently submitted to both MASCOT and SEQUEST analysis for sequence assignment. All proteins identified from at least one unique peptide sequence regardless of values were then submitted to Scaffold Q+ with the 384 tagged spectra from the MS analysis to determine relative quantification. After intensity based normalization the technical replicates were pooled and relative quantity of each identified protein was compared to the related mock treatment of the same biological replicate. Proteins with expression greater than or equal to |±1.5| fold change within a biological replicate were verified using the Mann-Whitney test (p-value < 0.05). Proteins were only considered differentially expressed if in at least two biological replicates they had |±1.5| fold change and p-value < 0.05. This resulted in a list of differentially expressed proteins for each of the 4 treatments (see Tables 1 and 2).
